# TagSmart: analysis and visualization for yeast mutant fitness data measured by tag microarrays

**DOI:** 10.1186/1471-2105-8-128

**Published:** 2007-04-18

**Authors:** Chulyun Kim, Sangkyum Kim, Russell Dorer, Dan Xie, Jiawei Han, Sheng Zhong

**Affiliations:** 1 Department Computer Science, University of Illinois at Urbana Champaign, Urbana, IL, USA; 2 Department of Bioengineering, University of Illinois at Urbana Champaign, Urbana, IL, USA; 3 Department of Pathology, Virginia Mason Medical Center, 1100 Ninth Avenue, Seattle, WA, USA; 4 Department of Statistics, University of Illinois at Urbana Champaign, Champaign, IL, USA; 5 Institute for Genomic Biology, University of Illinois at Urbana Champaign, Urbana, IL, USA; 6 School of Electrical Engineering and Computer Science, Seoul National University, South Korea

## Abstract

**Background:**

A nearly complete collection of gene-deletion mutants (96% of annotated open reading frames) of the yeast *Saccharomyces cerevisiae *has been systematically constructed. Tag microarrays are widely used to measure the fitness of each mutant in a mutant mixture. The tag array experiments can have a complex experimental design, such as time course measurements and drug treatment with multiple dosages.

**Results:**

TagSmart is a web application for analysis and visualization of *Saccharomyces cerevisiae *mutant fitness data measured by tag microarrays. It implements a robust statistical approach to assess the concentration differences among S. cerevisiae mutant strains. It also provides an interactive environment for data analysis and visualization. TagSmart has the following advantages over previously described analysis procedures: 1) it is user-friendly software rather than merely a description of analytical procedure; 2) It can handle complicated experimental designs, such as multiple time points and treatment with multiple dosages; 3) it has higher sensitivity and specificity; 4) It allows users to mask out "bad" tags in the analysis.

Two biological tests were performed to illustrate the performance of TagSmart. First, we generated titration mixtures of mutant strains, in which the relative concentration of each strain was controlled. We used tag microarrays to measure the numbers of tag copies in each titration mixture. The data was analyzed with TagSmart and the result showed high precision and recall. Second, TagSmart was applied to a dataset in which heterozygous deletion strain mixture pools were treated with a new drug, Cincreasin. TagSmart identified 53 mutant strains as sensitive to Cincreasin treatment. We individually tested each identified mutant, and found 52 out of the 53 predicted mutants were indeed sensitive to Cincreasin.

**Conclusion:**

TagSmart is provided "as is" to analyze tag array data produced by Affymetrix and Agilent arrays. TagSmart web application is assessable by Windows, Mac, and Linux users. It also has a downloadable version for execution on PCs running Windows. TagSmart is available for academic use at:

## 1. Background

A nearly complete collection of gene-deletion mutants (96% of annotated open reading frames) of the yeast *Saccharromyces cerevisiae *has been systematically constructed [1,2]. Each deletion is marked with two unique oligonucleotide tags, making it possible to use microarrays and the tag arrays [3] to measure the relative abundance of each mutant strain in a mixture. The relative fitness of every individual gene deletion mutant can be compared across multiple intercellular environments. A general question of interest is which gene-deletion and environment interaction is most lethal or most viable.

Every mutant is barcoded with two tags, namely the uptag and the downtag. The two tags are deletion-specific. They are synthesized into the genome of the deletion strain at the location of the deleted gene. For Affymetrix tag arrays, four probes on the microarray are designed to hybridize to the sense and antisense strands of each tag. These probe sets are indicated by Perfect Match (PM), Mis-Match (MM), complementary Perfect Match (cPM), and complementary Mis-Match (cMM). In summary, every mutant strain is represented by eight probe readouts: uptag-PM, uptag-cPM, uptag-MM, uptag-cMM, downtag-PM, downtag-cPM, downtag-MM, downtag-cMM [Additional file 1]. Please refer to [4] for details of Agilent tag arrays.

A general experiment design is a two-environment, multiple-time-point design [1,2,5]. Two mixtures of gene-deletion mutants are grown under two different environmental conditions: a drug-treated condition and a control condition. Mutant samples are collected from both collections at a series of time points, e.g., 4, 8, and 16 cell generations. DNA of these samples are retrieved, amplified, and hybridized to tag arrays. We summarize the experimental designs and analytical procedures in published literature at below and in Table 1.

**Table 1:** Summary of experimental design and data structure in previous studies.

	Multiple time points	Multiple treatment dosages	Replicates in treatment	Replicates in control
Winzeler et al [1]	Y	N	N	N
Ooi et al [15]	N	N	Y	Y
Ooi et al [16]	N	N	Y	Y
Giaever et al [2]	Y	N	N	Y
Warren et al [17]	N	N	Y	Y
Lee et al [18]	N	N	Y	Y
Pan et al [19]	N	Y	Y	Y
Lum et al [6]	N	N	N	Y
Birrell et al [20]	N	N	Y	Y
Dorer et al [5]	Y	Y	Y	Y
Yuan et al [4]	N	N	Y	Y
Peyser et al [21]	N	N	Y	Y

A few analytical procedures have been proposed to analyze tag microarray data. (see Additional file 2 for a detailed review of these procedures.) Most of these procedures were designed to handle a specific dataset generated by a specific experiment, and therefore are not applicable to analyzing other data generated from a different experimental design, with exceptions to the procedure described by Giaever et al. [2] and the procedure we recently proposed [5]. The latter procedure is more general than the Giaever procedure because it can handle treatment with multiple dosages. The TagSmart software implements the latter procedure (hereafter referred to as the TagSmart procedure). When there is a complex experimental design (e.g., multiple time points, or multiple dosages), TagSmart will take the most advantage of the comprehensive data available. When data is generated from a simple experimental design, the TagSmart procedure will automatically degenerate into a simpler procedure.

## 2. Implementation

### 2.1 TagSmart software

TagSmart [3] is a web application that can be operated by web browsers, such as Mozilla Firefox, Safari, and Internet Explorer. TagSmart also has a downloadable version for execution on PCs running on Windows. TagSmart has three modules: data preprocessing, computation, and visualization.

#### Data preprocessing module

The data preprocessing module integrates data files and annotation files into one easily interpretable data file. The following files are required as input files to the preprocessing module: 1) a series of tag array data files, in either CEL or TXT format; 2) a chip description file (CDF file); 3) an array description file; 4) a tag mask file; and 5) a user-supplied experiment description file. Except that the tag array data files and experiment description file should be provided by the user, all the other files can be downloaded from the TagSmart website. The CEL format data files are direct outputs of an Affymetrix scanner. If users have applied Affymetrix software such as GCOS [3] to process the data, they may have data files in TXT format. TagSmart allows users to supply data files in either CEL or TXT format. The CDF file records the coordinates of each tag on the tag microarray. The array description file links each tag to its corresponding open reading frame and gene name. Because the same tag was used to make both the homozygous deletion mutant [1] and the heterozygous deletion mutant [6], the analysis of heterozygous and homozygous mutants share the same array description file (the analysis of homozygous mutants only uses a subset of this file). The tag mask file records a list of tags that do not show "responsiveness" to the concentration change of their corresponding mutants. These tags are considered to be bad (the procedure of detecting bad tags will be described later). The user can choose to mask out the "bad" tags in the subsequent analysis by clicking on the "Bad tag filtering" checkbox (Additional file 3). Finally, the experiment description file is a user-supplied file, recording the experimental condition (e.g., treated/control, dosage, time, etc.) for each array data file. Users should follow the instruction on the TagSmart help-page to construct this file. The output of the preprocessing module is an easily interpretable data file in tabular format. Instead of averaging the multiple signals of a mutant (e.g., four PM signals in the Affymetrix platform), the preprocessing module retains them individually in the output.

#### Computation module

By choosing the radio button of "Analyze a preprocessed data file" in the main page, users activate the computation module. Users should specify desired criteria for selecting mutants in the subsequent webpage (Additional file 4). Fold Change (FC) and Q-value (equivalent to false discovery rate) are allowed. If the user has array data for a common pool of mutant mixture, such as a time 0 sample before the separation of mutant growth in treatment and control, she/he can choose to use such data to get more precise estimate of mutant growth rates. This is achieved by checking the "Generation-0 correction" check box (The procedure to handle a common mixture pool will be described later). Advanced users are also allowed to tune a parameter called the number of permutations. As in the Statistical Procedure section described below, TagSmart employs a matched permutation method to obtain background distribution. The number of permutation is positively correlated with the accuracy of computation but also computation time. We suggest a default number of 500 permutations, which is a balance between accuracy and time. Additional file 5 gives an example text output of a computation. Mutants that satisfied the user-defined thresholds are listed. Their related information, including open reading frame's name (ORF), gene name, the two associated tags, p-value, q-value, and fold change are provided. Users can sort the output by any information with a click on the corresponding column name. A more comprehensive report, including the actual data and experimental conditions, can be saved as a text file by clicking the disk icon on the output webpage.

#### Visualization module

**Figure 1:**
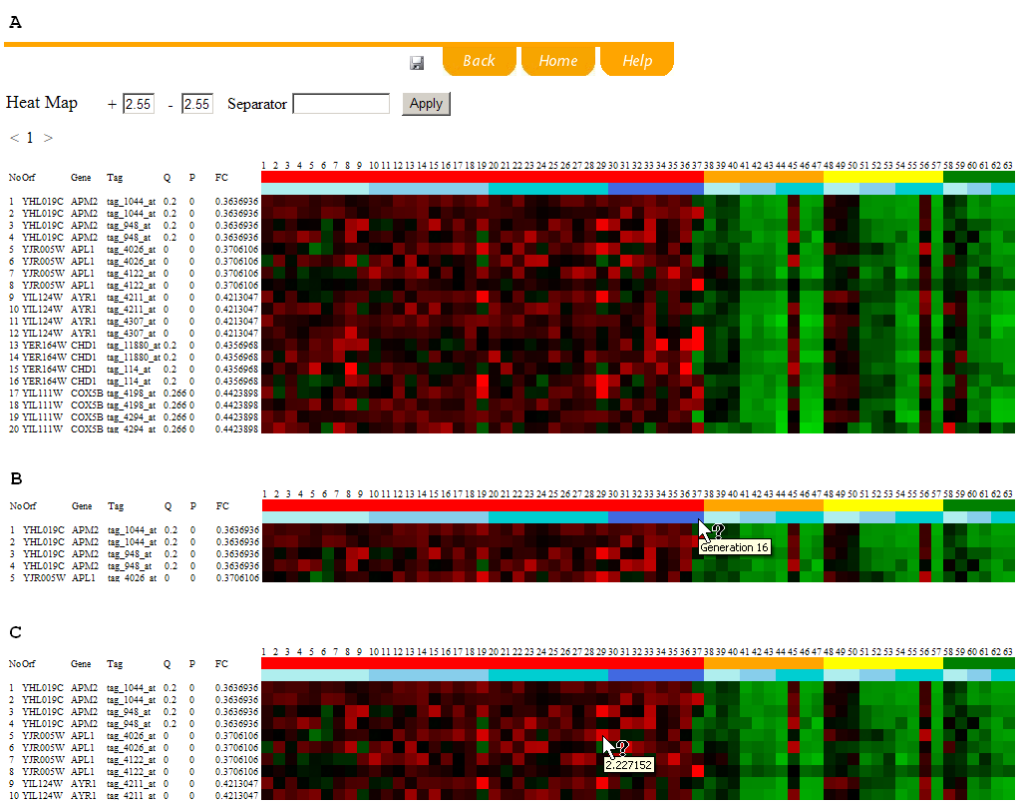
(A) An example TagSmart's graphical output. Every mutant is represented in 4 rows, representing signals from four tag-probe pairs (uptag-PM, uptag-cPM, downtag-PM, and downtag-cPM). The arrays are sequentially marked on the top row. The first two colored lines indicate the treatment and the time under which the mutant mixture was harvested and DNA was retrieved. The heatmap starts from the third colored line. Red indicates good fitness in treatment, and green indicates reduced fitness in treatment. (B) When mouse cursor is moved over the first two colored lines, the corresponding information for treatment and time will appear on screen. (C) When mouse cursor is over a dot in the heatmap, the actual fitness value will appear.

An interactive graphical display of the computation result is accessible by clicking the "heatmap" icon after the computation (Figure 1A). Alternatively, the saved report file on a user's local computer can be uploaded onto the server and visualized using the visualization module. TagSmart adopts heatmap as the way to present mutant's relative concentration. The first two rows in the heatmap use a novel color scheme to represent experimental design information. The first row represents the treatment factor (treatment 0, treatment 1, etc.). The second row represents the time factor (Generation 0, Generation 4, etc.). From the third row on, a traditional heatmap is applied to show the relative concentration of each mutant. Red represents higher concentration and green represents lower concentration. Detailed information on treatment, time, and array signal can be monitored by moving mouse cursor over the corresponding color-coded region (Figure 1B and 1C).

We tested TagSmart with multiple web browsers under Linux, MacOS, and Windows operation systems. TagSmart is implemented with C# programming language and ASP.NET technology. TagSmart is currently hosted on a Dell rack server with dual 3 GHz Intel(R) Xeon(TM) dual-core processors and 6GB RAM. A standalone executable for Windows is also downloadable from the TagSmart website.

### 2.2. Statistical Procedure

We describe TagSmart's statistical procedure assuming data comes from the most complicated setting (i.e., multiple time points and treatment with multiple dosages). This procedure automatically degenerates into a simpler procedure when data comes from a simpler experimental design.

Array signals are first normalized to make 1% trimmed mean the same across all arrays [7]. Let *y*_*iαβγδτ *_be the normalized signal for mutant i, in environmental condition *α*, at time point *β*, measured by tag *γ *(*uptag *and *downtag*) and probe *δ *(*PM *and *cPM*), on the replicate array *τ*. For notational simplicity, we will suppress the mutant indicator *i *hereafter. When time-0 data is available, users can use the following metric to represent the growth rate measured by a probe at time *β*: xαβγδτ=yαβγδτ−yβ=0,γδ¯yβ=0,γδ¯
 MathType@MTEF@5@5@+=feaafiart1ev1aaatCvAUfKttLearuWrP9MDH5MBPbIqV92AaeXatLxBI9gBaebbnrfifHhDYfgasaacH8akY=wiFfYdH8Gipec8Eeeu0xXdbba9frFj0=OqFfea0dXdd9vqai=hGuQ8kuc9pgc9s8qqaq=dirpe0xb9q8qiLsFr0=vr0=vr0dc8meaabaqaciaacaGaaeqabaqabeGadaaakeaacqWG4baEdaWgaaWcbaacciGae8xSdeMae8NSdiMae83SdCMae8hTdqMae8hXdqhabeaakiabg2da9maalaaabaGaemyEaK3aaSbaaSqaaiab=f7aHjab=j7aIjab=n7aNjab=r7aKjab=r8a0bqabaGccqGHsisldaqdaaqaaiabdMha5naaBaaaleaacqWFYoGycqGH9aqpcqaIWaamcqGGSaalcqWFZoWzcqWF0oazaeqaaaaaaOqaamaanaaabaGaemyEaK3aaSbaaSqaaiab=j7aIjabg2da9iabicdaWiabcYcaSiab=n7aNjab=r7aKbqabaaaaaaaaaa@5569@, where yβ=0,γδ¯
 MathType@MTEF@5@5@+=feaafiart1ev1aaatCvAUfKttLearuWrP9MDH5MBPbIqV92AaeXatLxBI9gBaebbnrfifHhDYfgasaacH8akY=wiFfYdH8Gipec8Eeeu0xXdbba9frFj0=OqFfea0dXdd9vqai=hGuQ8kuc9pgc9s8qqaq=dirpe0xb9q8qiLsFr0=vr0=vr0dc8meaabaqaciaacaGaaeqabaqabeGadaaakeaadaqdaaqaaiabdMha5naaBaaaleaaiiGacqWFYoGycqGH9aqpcqaIWaamcqGGSaalcqWFZoWzcqWF0oazaeqaaaaaaaa@3622@ is the average across all array replicates at time-0. The growth rate measurement *x*_*αβγδτ *_was inspired by the Coefficient of Variation (CV) statistic. With the observation that signals with larger magnitude usually have larger variability, *x*_*αβγδτ *_can be regarded as a signal for concentration change, with the raw signal intensity penalized. When time-0 data is not available, the normalized signal is directly passed onto the next step: *x*_*αβγδτ *_= *y*_*αβγδτ*_.

To compare the difference of concentration changes between different experimental conditions, we first compute a modified T statistic:

T=xα=trt¯−xα=contr¯s+s0
 MathType@MTEF@5@5@+=feaafiart1ev1aaatCvAUfKttLearuWrP9MDH5MBPbIqV92AaeXatLxBI9gBaebbnrfifHhDYfgasaacH8akY=wiFfYdH8Gipec8Eeeu0xXdbba9frFj0=OqFfea0dXdd9vqai=hGuQ8kuc9pgc9s8qqaq=dirpe0xb9q8qiLsFr0=vr0=vr0dc8meaabaqaciaacaGaaeqabaqabeGadaaakeaacqWGubavcqGH9aqpdaWcaaqaamaanaaabaGaemiEaG3aaSbaaSqaaGGaciab=f7aHjabg2da9iabdsha0jabdkhaYjabdsha0bqabaaaaOGaeyOeI0Yaa0aaaeaacqWG4baEdaWgaaWcbaGae8xSdeMaeyypa0Jaem4yamMaem4Ba8MaemOBa4MaemiDaqNaemOCaihabeaaaaaakeaacqWGZbWCcqGHRaWkcqWGZbWCdaWgaaWcbaGaeGimaadabeaaaaaaaa@48CE@

where xα=trt¯
 MathType@MTEF@5@5@+=feaafiart1ev1aaatCvAUfKttLearuWrP9MDH5MBPbIqV92AaeXatLxBI9gBaebbnrfifHhDYfgasaacH8akY=wiFfYdH8Gipec8Eeeu0xXdbba9frFj0=OqFfea0dXdd9vqai=hGuQ8kuc9pgc9s8qqaq=dirpe0xb9q8qiLsFr0=vr0=vr0dc8meaabaqaciaacaGaaeqabaqabeGadaaakeaadaqdaaqaaiabdIha4naaBaaaleaaiiGacqWFXoqycqGH9aqpcqWG0baDcqWGYbGCcqWG0baDaeqaaaaaaaa@355D@ and xα=contr¯
 MathType@MTEF@5@5@+=feaafiart1ev1aaatCvAUfKttLearuWrP9MDH5MBPbIqV92AaeXatLxBI9gBaebbnrfifHhDYfgasaacH8akY=wiFfYdH8Gipec8Eeeu0xXdbba9frFj0=OqFfea0dXdd9vqai=hGuQ8kuc9pgc9s8qqaq=dirpe0xb9q8qiLsFr0=vr0=vr0dc8meaabaqaciaacaGaaeqabaqabeGadaaakeaadaqdaaqaaiabdIha4naaBaaaleaaiiGacqWFXoqycqGH9aqpcqWGJbWycqWGVbWBcqWGUbGBcqWG0baDcqWGYbGCaeqaaaaaaaa@3807@ are average concentration changes under treated and untreated conditions, respectively. If the treatment was conducted with multiple dosages, xα=trt¯
 MathType@MTEF@5@5@+=feaafiart1ev1aaatCvAUfKttLearuWrP9MDH5MBPbIqV92AaeXatLxBI9gBaebbnrfifHhDYfgasaacH8akY=wiFfYdH8Gipec8Eeeu0xXdbba9frFj0=OqFfea0dXdd9vqai=hGuQ8kuc9pgc9s8qqaq=dirpe0xb9q8qiLsFr0=vr0=vr0dc8meaabaqaciaacaGaaeqabaqabeGadaaakeaadaqdaaqaaiabdIha4naaBaaaleaaiiGacqWFXoqycqGH9aqpcqWG0baDcqWGYbGCcqWG0baDaeqaaaaaaaa@355D@ is computed with all the data from all dosages. *s *is the pooled standard deviation of all data. It is computed by:

s=((1n1+1n2)×{∑α=trt,β,γδτ[xα=trt,β,γδτ−xα=trt¯]2+∑α=contr,β,γδτ[xα=contr,β,γδτ−xα=contr¯]2}n1+n2−2)12
 MathType@MTEF@5@5@+=feaafiart1ev1aaatCvAUfKttLearuWrP9MDH5MBPbIqV92AaeXatLxBI9gBaebbnrfifHhDYfgasaacH8akY=wiFfYdH8Gipec8Eeeu0xXdbba9frFj0=OqFfea0dXdd9vqai=hGuQ8kuc9pgc9s8qqaq=dirpe0xb9q8qiLsFr0=vr0=vr0dc8meaabaqaciaacaGaaeqabaqabeGadaaakeaacqWGZbWCcqGH9aqpdaqadaqaamaalaaabaWaaeWaaeaadaWcaaqaaiabigdaXaqaaiabd6gaUnaaBaaaleaacqaIXaqmaeqaaaaakiabgUcaRmaalaaabaGaeGymaedabaGaemOBa42aaSbaaSqaaiabikdaYaqabaaaaaGccaGLOaGaayzkaaGaey41aqRaei4EaS3aaabuaeaacqGGBbWwcqWG4baEdaWgaaWcbaacciGae8xSdeMaeyypa0JaemiDaqNaemOCaiNaemiDaqNaeiilaWIae8NSdiMaeiilaWIae83SdCMae8hTdqMae8hXdqhabeaakiabgkHiTmaanaaabaGaemiEaG3aaSbaaSqaaiab=f7aHjabg2da9iabdsha0jabdkhaYjabdsha0bqabaaaaOGaeiyxa01aaWbaaSqabeaacqaIYaGmaaaabaGae8xSdeMaeyypa0JaemiDaqNaemOCaiNaemiDaqNaeiilaWIae8NSdiMaeiilaWIae83SdCMae8hTdqMae8hXdqhabeqdcqGHris5aOGaey4kaSYaaabuaeaacqGGBbWwcqWG4baEdaWgaaWcbaGae8xSdeMaeyypa0Jaem4yamMaem4Ba8MaemOBa4MaemiDaqNaemOCaiNaeiilaWIae8NSdiMaeiilaWIae83SdCMae8hTdqMae8hXdqhabeaakiabgkHiTmaanaaabaGaemiEaG3aaSbaaSqaaiab=f7aHjabg2da9iabdogaJjabd+gaVjabd6gaUjabdsha0jabdkhaYbqabaaaaOGaeiyxa01aaWbaaSqabeaacqaIYaGmaaaabaGae8xSdeMaeyypa0Jaem4yamMaem4Ba8MaemOBa4MaemiDaqNaemOCaiNaeiilaWIae8NSdiMaeiilaWIae83SdCMae8hTdqMae8hXdqhabeqdcqGHris5aOGaeiyFa0habaGaemOBa42aaSbaaSqaaiabigdaXaqabaGccqGHRaWkcqWGUbGBdaWgaaWcbaGaeGOmaidabeaakiabgkHiTiabikdaYaaaaiaawIcacaGLPaaadaahaaWcbeqaamaalaaabaGaeGymaedabaGaeGOmaidaaaaaaaa@B184@

where ∑α=trt
 MathType@MTEF@5@5@+=feaafiart1ev1aaatCvAUfKttLearuWrP9MDH5MBPbIqV92AaeXatLxBI9gBaebbnrfifHhDYfgasaacH8akY=wiFfYdH8Gipec8Eeeu0xXdbba9frFj0=OqFfea0dXdd9vqai=hGuQ8kuc9pgc9s8qqaq=dirpe0xb9q8qiLsFr0=vr0=vr0dc8meaabaqaciaacaGaaeqabaqabeGadaaakeaadaaeqbqaaaWcbaacciGae8xSdeMaeyypa0JaemiDaqNaemOCaiNaemiDaqhabeqdcqGHris5aaaa@35CA@ denotes sum over all treated signals. If there are multiple dosages, all these dosages should be summed. *n*_1 _and *n*_2 _are the numbers of *x *s under treated and untreated conditions, respectively. s_0 _is a small positive constant (0.001) that ensures T not outrageously large.

**Figure 2:**
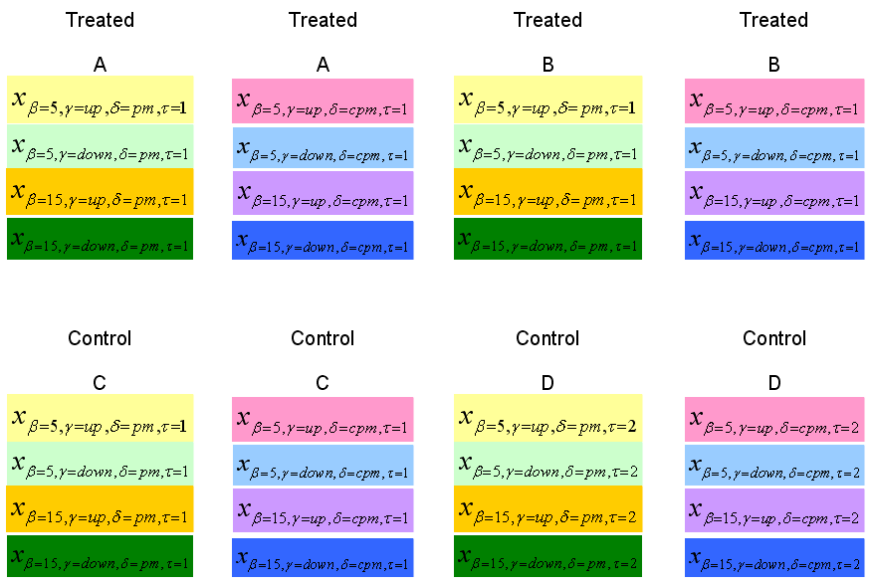
Data table for a mutant in a hypothetical experiment. This hypothetical experiment has two replicate mutant mixtures in control condition, and two treated mixtures under different treatment dosage. Mutant samples were harvested at both cell generations 5 and 15. Eight tag arrays should be used to gather the data. TagSmart's matched permutation procedure switches the signals with the same color.

We use a matched permutation strategy to generate background distribution for the T statistic. To illustrate the matched permutation procedure, we assume that data comes from the following hypothetical experiment. Let A and B denote two mutant mixtures treated with high and low dosages of a drug, respectively. Mutant mixtures C and D are replicates grown under control condition. Mutant samples are collected at 5 and 15 cell generations. In this hypothetical experiment, 8 tag arrays are used to gather data. We permute *x*_*αβγδτ *_with the same time (*β*), tag (*γ*), and probe (*δ*). To see this permutation procedure graphically, signals are arranged in Figure 2 and color coded. The signals are permutated under the constraint that a signal can only be switched to a box with the same color as its original one.

With K permutations, we obtain K new statistics T^k^, k = 1, 2, ..., K. We compute a q-value (roughly equivalent to false discovery rate [8]) for every mutant. The statistical interpretation for a mutant's q-value is: if the threshold is set so that this mutant is the last mutant to be called significant, the q-value is the estimated percentage of false positives among all the mutants being called significant. We compute the q-value for a mutant by [9,10]:

q^=1K∑k=1K∑j=1NI{|Tjk| ≥ |T|}max⁡{1,∑j=1NI{|Tj| ≥ |T|}π^0(c0)
 MathType@MTEF@5@5@+=feaafiart1ev1aaatCvAUfKttLearuWrP9MDH5MBPbIqV92AaeXatLxBI9gBaebbnrfifHhDYfgasaacH8akY=wiFfYdH8Gipec8Eeeu0xXdbba9frFj0=OqFfea0dXdd9vqai=hGuQ8kuc9pgc9s8qqaq=dirpe0xb9q8qiLsFr0=vr0=vr0dc8meaabaqaciaacaGaaeqabaqabeGadaaakeaacuWGXbqCgaqcaiabg2da9maalaaabaWaaSaaaeaacqaIXaqmaeaacqWGlbWsaaWaaabCaeaadaaeWbqaaiabdMeajjabcUha7jabcYha8jabdsfaunaaDaaaleaacqWGQbGAaeaacqWGRbWAaaGccqGG8baFcqqGGaaicqGHLjYScqqGGaaicqGG8baFcqWGubavcqGG8baFaSqaaiabdQgaQjabg2da9iabigdaXaqaaiabd6eaobqdcqGHris5aOGaeiyFa0haleaacqWGRbWAcqGH9aqpcqaIXaqmaeaacqWGlbWsa0GaeyyeIuoaaOqaaiGbc2gaTjabcggaHjabcIha4jabcUha7jabigdaXiabcYcaSmaaqahabaGaemysaKKaei4EaSNaeiiFaWNaemivaq1aaSbaaSqaaiabdQgaQbqabaGccqGG8baFcqqGGaaicqGHLjYScqqGGaaicqGG8baFcqWGubavcqGG8baFaSqaaiabdQgaQjabg2da9iabigdaXaqaaiabd6eaobqdcqGHris5aOGaeiyFa0haaGGaciqb=b8aWzaajaWaaSbaaSqaaiabicdaWaqabaGccqGGOaakcqWGJbWydaWgaaWcbaGaeGimaadabeaakiabcMcaPaaa@785D@, where j = 1, 2, ..., N is the index for mutants. T is the T-statistic computed from un-permuted data. π^0(c0)
 MathType@MTEF@5@5@+=feaafiart1ev1aaatCvAUfKttLearuWrP9MDH5MBPbIqV92AaeXatLxBI9gBaebbnrfifHhDYfgasaacH8akY=wiFfYdH8Gipec8Eeeu0xXdbba9frFj0=OqFfea0dXdd9vqai=hGuQ8kuc9pgc9s8qqaq=dirpe0xb9q8qiLsFr0=vr0=vr0dc8meaabaqaciaacaGaaeqabaqabeGadaaakeaaiiGacuWFapaCgaqcamaaBaaaleaacqaIWaamaeqaaOGaeiikaGIaem4yam2aaSbaaSqaaiabicdaWaqabaGccqGGPaqkaaa@33C9@ is the estimated proportion of mutants with no fitness difference between experimental conditions. π^0(c0)
 MathType@MTEF@5@5@+=feaafiart1ev1aaatCvAUfKttLearuWrP9MDH5MBPbIqV92AaeXatLxBI9gBaebbnrfifHhDYfgasaacH8akY=wiFfYdH8Gipec8Eeeu0xXdbba9frFj0=OqFfea0dXdd9vqai=hGuQ8kuc9pgc9s8qqaq=dirpe0xb9q8qiLsFr0=vr0=vr0dc8meaabaqaciaacaGaaeqabaqabeGadaaakeaaiiGacuWFapaCgaqcamaaBaaaleaacqaIWaamaeqaaOGaeiikaGIaem4yam2aaSbaaSqaaiabicdaWaqabaGccqGGPaqkaaa@33C9@ is estimated by [9,10]:

${\hat{\pi }}_{0}(c_{0})=\frac{\frac{1}{N}\sum_{j=1}^{N}I\{|T_{j}|<c_{0}\}}{\frac{1}{NK}\sum_{k=1}^{K}\sum_{j=1}^{N}I\{|T_{j}^{k}|<c_{0}\}}$, and C_0 _is a pre-defined constant (0.5). The choice of C_0 _does not affect the estimate of π^0(c0)
 MathType@MTEF@5@5@+=feaafiart1ev1aaatCvAUfKttLearuWrP9MDH5MBPbIqV92AaeXatLxBI9gBaebbnrfifHhDYfgasaacH8akY=wiFfYdH8Gipec8Eeeu0xXdbba9frFj0=OqFfea0dXdd9vqai=hGuQ8kuc9pgc9s8qqaq=dirpe0xb9q8qiLsFr0=vr0=vr0dc8meaabaqaciaacaGaaeqabaqabeGadaaakeaaiiGacuWFapaCgaqcamaaBaaaleaacqaIWaamaeqaaOGaeiikaGIaem4yam2aaSbaaSqaaiabicdaWaqabaGccqGGPaqkaaa@33C9@ as long as C_0 _is reasonably small [8,9].

Finally, the fold change (FC) between treatment and control is computed by:

FC=∑βaβ×FCβ
 MathType@MTEF@5@5@+=feaafiart1ev1aaatCvAUfKttLearuWrP9MDH5MBPbIqV92AaeXatLxBI9gBaebbnrfifHhDYfgasaacH8akY=wiFfYdH8Gipec8Eeeu0xXdbba9frFj0=OqFfea0dXdd9vqai=hGuQ8kuc9pgc9s8qqaq=dirpe0xb9q8qiLsFr0=vr0=vr0dc8meaabaqaciaacaGaaeqabaqabeGadaaakeaacqWGgbGrcqWGdbWqcqGH9aqpdaaeqbqaaiabdggaHnaaBaaaleaaiiGacqWFYoGyaeqaaaqaaiab=j7aIbqab0GaeyyeIuoakiabgEna0kabdAeagjabdoeadnaaBaaaleaacqWFYoGyaeqaaaaa@3CB6@. It is a weighted sum of each time point fold change. We require ∑βaβ=1
 MathType@MTEF@5@5@+=feaafiart1ev1aaatCvAUfKttLearuWrP9MDH5MBPbIqV92AaeXatLxBI9gBaebbnrfifHhDYfgasaacH8akY=wiFfYdH8Gipec8Eeeu0xXdbba9frFj0=OqFfea0dXdd9vqai=hGuQ8kuc9pgc9s8qqaq=dirpe0xb9q8qiLsFr0=vr0=vr0dc8meaabaqaciaacaGaaeqabaqabeGadaaakeaadaaeqbqaaiabdggaHnaaBaaaleaaiiGacqWFYoGyaeqaaOGaeyypa0JaeGymaedaleaacqWFYoGyaeqaniabggHiLdaaaa@358A@. Larger *a*_*β *_will stress the importance of that *β *time point. *FC*_*β *_is the fold change at time *β*. It is defined as:

FCβ=1#(γ,δ)∑γ∑δ∑α=trt∑τxαβγδτ∑α=contr∑τxαβγδτ
 MathType@MTEF@5@5@+=feaafiart1ev1aaatCvAUfKttLearuWrP9MDH5MBPbIqV92AaeXatLxBI9gBaebbnrfifHhDYfgasaacH8akY=wiFfYdH8Gipec8Eeeu0xXdbba9frFj0=OqFfea0dXdd9vqai=hGuQ8kuc9pgc9s8qqaq=dirpe0xb9q8qiLsFr0=vr0=vr0dc8meaabaqaciaacaGaaeqabaqabeGadaaakeaacqWGgbGrcqWGdbWqdaWgaaWcbaacciGae8NSdigabeaakiabg2da9maalaaabaGaeGymaedabaGaei4iamIaeiikaGIae83SdCMaeiilaWIae8hTdqMaeiykaKcaamaaqafabaWaaabuaeaadaWcaaqaamaaqafabaWaaabuaeaacqWG4baEdaWgaaWcbaGae8xSdeMae8NSdiMae83SdCMae8hTdqMae8hXdqhabeaaaeaacqWFepaDaeqaniabggHiLdaaleaacqWFXoqycqGH9aqpcqWG0baDcqWGYbGCcqWG0baDaeqaniabggHiLdaakeaadaaeqbqaamaaqafabaGaemiEaG3aaSbaaSqaaiab=f7aHjab=j7aIjab=n7aNjab=r7aKjab=r8a0bqabaaabaGae8hXdqhabeqdcqGHris5aaWcbaGae8xSdeMaeyypa0Jaem4yamMaem4Ba8MaemOBa4MaemiDaqNaemOCaihabeqdcqGHris5aaaaaSqaaiab=r7aKbqab0GaeyyeIuoaaSqaaiab=n7aNbqab0GaeyyeIuoaaaa@7130@

where *α, β, γ, δ*, and *τ *are defined the same as above. It is worth noticing that TagSmart does not first average all probe signals and then take the ratio, but rather it first takes ratio on the same probe and then averages over all tags and probes. TagSmart jointly uses q-value and FC to call significant mutants.

## 3. Results

### Titration Experiment

**Figure 3:**
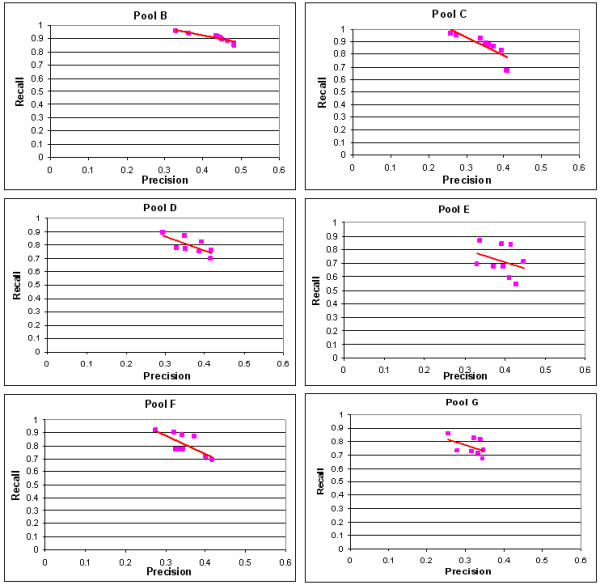
Precision vs. Recall for TagSmart. The six panels represent the mutant mixture pools B-F, respectively. For a wide range of thresholds, the precision and the recall from TagSmart are plotted, and a linear regression line is fitted.

To illustrate TagSmart's performance, we did a titration experiment using homozygous deletion mutants. Eight mutant mixture pools were made, which were denoted as pools A, B, C, D, E, F and G, respectively. The mutants had roughly equal concentrations in mixture pools A and G. One sixth of the mutants were diluted into 1/25 concentration whereas the concentration of the rest mutants were untouched in pool B. Another one sixth, not overlapping with the first one sixth, were diluted to 1/25 concentration in pool C, so did pools D, E, and F. In the end pools B to F each had one sixth of the mutants diluted. DNA from each mutant pool was hybridized to a tag microarray. TagSmart procedure was applied to identify the mutants with lower concentration in pools C to G. A wide range of thresholds for determining the mutants with lower concentration were applied, and for each threshold the computationally identified mutants were compared to the real diluted mutants. We computed the precision and the recall of TagSmart procedure (Figure 3). Precision and recall are defined as follows.

Precision ={True positive} ∩ { Predicted positive }{ Predicted positive }
 MathType@MTEF@5@5@+=feaafiart1ev1aaatCvAUfKttLearuWrP9MDH5MBPbIqV92AaeXatLxBI9gBaebbnrfifHhDYfgasaacH8akY=wiFfYdH8Gipec8Eeeu0xXdbba9frFj0=OqFfea0dXdd9vqai=hGuQ8kuc9pgc9s8qqaq=dirpe0xb9q8qiLsFr0=vr0=vr0dc8meaabaqaciaacaGaaeqabaqabeGadaaakeaacqqGqbaucqqGYbGCcqqGLbqzcqqGJbWycqqGPbqAcqqGZbWCcqqGPbqAcqqGVbWBcqqGUbGBcqqGGaaicqGH9aqpdaWcaaqaaiabbUha7jabbsfaujabbkhaYjabbwha1jabbwgaLjabbccaGiabbchaWjabb+gaVjabbohaZjabbMgaPjabbsha0jabbMgaPjabbAha2jabbwgaLjabb2ha9jabbccaGiablMIijjabbccaGiabbUha7jabbccaGiabbcfaqjabbkhaYjabbwgaLjabbsgaKjabbMgaPjabbogaJjabbsha0jabbwgaLjabbsgaKjabbccaGiabbchaWjabb+gaVjabbohaZjabbMgaPjabbsha0jabbMgaPjabbAha2jabbwgaLjabbccaGiabb2ha9bqaaiabbUha7jabbccaGiabbcfaqjabbkhaYjabbwgaLjabbsgaKjabbMgaPjabbogaJjabbsha0jabbwgaLjabbsgaKjabbccaGiabbchaWjabb+gaVjabbohaZjabbMgaPjabbsha0jabbMgaPjabbAha2jabbwgaLjabbccaGiabb2ha9baaaaa@8A42@

Recall ={True positive} ∩ { Predicted positive }{ True positive }
 MathType@MTEF@5@5@+=feaafiart1ev1aaatCvAUfKttLearuWrP9MDH5MBPbIqV92AaeXatLxBI9gBaebbnrfifHhDYfgasaacH8akY=wiFfYdH8Gipec8Eeeu0xXdbba9frFj0=OqFfea0dXdd9vqai=hGuQ8kuc9pgc9s8qqaq=dirpe0xb9q8qiLsFr0=vr0=vr0dc8meaabaqaciaacaGaaeqabaqabeGadaaakeaacqqGsbGucqqGLbqzcqqGJbWycqqGHbqycqqGSbaBcqqGSbaBcqqGGaaicqGH9aqpdaWcaaqaaiabbUha7jabbsfaujabbkhaYjabbwha1jabbwgaLjabbccaGiabbchaWjabb+gaVjabbohaZjabbMgaPjabbsha0jabbMgaPjabbAha2jabbwgaLjabb2ha9jabbccaGiablMIijjabbccaGiabbUha7jabbccaGiabbcfaqjabbkhaYjabbwgaLjabbsgaKjabbMgaPjabbogaJjabbsha0jabbwgaLjabbsgaKjabbccaGiabbchaWjabb+gaVjabbohaZjabbMgaPjabbsha0jabbMgaPjabbAha2jabbwgaLjabbccaGiabb2ha9bqaaiabbUha7jabbccaGiabbsfaujabbkhaYjabbwha1jabbwgaLjabbccaGiabbchaWjabb+gaVjabbohaZjabbMgaPjabbsha0jabbMgaPjabbAha2jabbwgaLjabbccaGiabb2ha9baaaaa@7F70@

Figure 3 shows that at the precision of 0.4, TagSmart achieves recalls of 0.7 to 0.9 in the titration data.

The titration experiment allows us to detect the "bad" tags that do not show consistent signal change for the diluted mutants. Each mutant is diluted in one of the eight mixture pools. The diluted concentration is 1/25 of the concentration of the undiluted concentration. We employed the following procedure to detect "bad" tags. For each tag, its signal from the diluted pool is compared to the average signal of this tag from the other seven undiluted pool (each mutant is only diluted in one of the eight pools). A tag is regarded as "bad" if its signal from the diluted pool is not smaller than its average signal from the undiluted pools. The "bad" tags are recorded into the tag mask file, which, by user's discretion, can be used to eliminate the bad tags from the subsequent analysis (see the preprocessing module). One reason for a tag being "bad" can attribute to the mutations of the synthetic DNA tags introduced during the construction of the deletion strains [11]. We note that a "bad" tag should not be taken literally, because there are many reasons that can contribute to inconsistency between the signal of a tag and the concentration change. For example, cross-hybridization to the probe on the array may contribute to the inconsistency.

### Cincreasin experiment

To illustrate the power of TagSmart in a real biological investigation, we applied TagSmart on a tag array dataset [5]. This dataset records the tag array measurements of heterozygous deletion mutants under four experimental conditions, including rich medium (control), 100, 200, and 400 uM treatment of a chemical called Cincreasin. Cincreasin is a newly synthesized molecule that inhibits the spindle checkpoint process by targeting Mps1 protein [5]. Additional file 6 summarizes this dataset. We restricted our analysis on 200 uM treatment data only.

TagSmart identified 53 mutants as sensitive to Cincreasin treatment (q-value = 1%, FC = 0.5, Additional file 7). Additional file 8 shows the fold changes of top 10 most sensitive mutants. Among theses mutants, Mps1 was shown to be the direct target of Cincreasin [5]. Mps1 is a dual-specificity kinase required for spindle pole body duplication and spindle checkpoint function [12]. Cincreasin blocks the spindle checkpoint response to a lack of tension on mitotic chromosome by inhibiting Mps1. However, the other mutants besides Mps1 being sensitive to Cincreasin might indicate that there is an aftermath of chained molecular responses to the inhibition of Mps1.

To validate these findings, we re-tested all the 53 heterozygous mutants individually with Cincreasin treatment in colonial growth assay (patch test). Mutant strains were grown in colonies in the same control environment as described in [5] and in 200 uM and 400 uM Cincreasin treated environments. Three wild type colonies were grown under each environment as negative controls. Cin8 homozygous deletion strain was used as positive control (sensitive to Cincreasin treatment), because Cincreasin has been previously shown to cause mis-segregation of chromosomes in cin8 null cells (Figure 3 in reference [5]). In this test, 52 out of the 53 predicted mutants showed significant sensitivity to Cincreasin in this test, comparing to three wild type colonies (Additional file 7 and Additional file 9). This high validation rate demonstrates TagSmart is very resistant to false positive reports. We recognize that due to the limited amount of mutant colonies we could test, it is infeasible for this test to address the amount of false negative reports. The titration experiment described previously in this paper did address both false positive and false negative reports.

The validated mutants fell into three classes: i) mutants with lesions in known components of the spindle, ii) mutants in genes of known function which lack any described role in chromosome segregation, and iii) mutants in genes of unknown function. The first functional category is highly relevant to the function of Cincreasin, an inhibitor of buddy yeast spindle checkout. It is worth further investigation whether the second class reflects additional molecular targets of Cincreasin that lie outside the spindle checkpoint, or previously undiscovered roles in spindle function for this class of genes.

## 4. Conclusion

Tag microarray data has inspired various research, including identification of gene function [1,13], identification of drug targets [5,6], and evolution and genetic robustness [14]. TagSmart is an interactive online software tool for the analysis of tag microarray data. It is freely available for non-commercial use at [3]. Our future work is to expand TagSmart for integrated analysis with other genomics data, such as expression data and double deletion mutant data.

## Availability and requirements

**Project name**: TagSmart

**Project home page**: 

**Operating systems**: Platform independent

**Programming language**: C#, ASP.NET

**Other requirements**: None

**License**: None

**Restrictions to use by non-academics**: licence needed

## Authors' contributions

SZ conceived the study, designed the analytical procedure, the software and the biological experiments. CK, SK and JH implemented the software. SZ, CK, SK and DX analyzed the data. RD performed the biological experiments. SZ coordinated the study and wrote the paper. All authors read and approved the final manuscript.

## Supplementary Material

Additional file 1 A generic gene-deletion cassette module. Supplementary figure 1 Click here for file

Additional file 2 Summary of previous methods. Supplementary document Click here for file

Additional file 3 Screenshot of the preprocessor module. Supplementary figure 2 Click here for file

Additional file 4 Screenshot of the computation module. Supplementary figure 3 Click here for file

Additional file 5 An example text output from TagSmart server. Supplementary figure 4 Click here for file

Additional file 6 Summary of the Cincreasin dataset. Supplementary table 1 Click here for file

Additional file 7 Summary of test results by colonial assays. Supplementary table 2 Click here for file

Additional file 8 Fold changes of the top 10 mutants sensitive to Cincreasin treatment. Supplementary figure 5 Click here for file

Additional file 9 Colonial assays for testing S.cerevisiae mutants in the presence of DMSO (control) and Cincreasin (treatment). Supplementary figure 6 Click here for file
