# Supplementary material for: TagSmart: analysis and visualization for yeast mutant fitness data measured by tag microarrays
Source: BMC Bioinformatics. 2007 Apr 18;8:128. doi: 10.1186/1471-2105-8-128 (PMC1868768; doi:10.1186/1471-2105-8-128)
Supplement: Additional file 1 — A generic gene-deletion cassette module. Supplementary figure 1 [file 1471-2105-8-128-S1.doc]

**Figure S1:** A generic gene-deletion cassette module. The biotin-labelled, deletion-specific primers (B-U1, B-U2-comp, B-D1 and B-D2-comp) are used to amplify the unique UPTAG and DNTAG sequences from genomic preparations generated in the fitness-profiling studies. B-U1 can hybridize to the uptag-PM and uptag-MM probes on the tag array. B-U2 can hybridize to the uptag-cPM and uptag-cMM probes, and so on. The figure is given by Giaever et al. [2][[1]](#footnote-2).


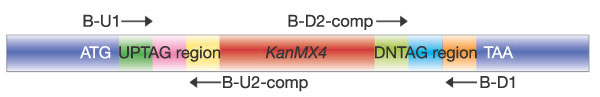


1. Reproduction permission is granted by Nature Publishing Group. [↑](#footnote-ref-2)
