# Supplementary material for: TagSmart: analysis and visualization for yeast mutant fitness data measured by tag microarrays
Source: BMC Bioinformatics. 2007 Apr 18;8:128. doi: 10.1186/1471-2105-8-128 (PMC1868768; doi:10.1186/1471-2105-8-128)
Supplement: Additional file 2 — Summary of previous methods. Supplementary document [file 1471-2105-8-128-S2.doc]

**Additional document**

Winzeler et al. fitted a simple linear model to the data of every mutant strain across a time course [1]. They regressed the log signal intensity to the population doubling time: Log_2_ (signal) = max(a + bt, 0) + e, where signal is the average PM reading of the two tags, t the doubling time, and e the error term. They regarded the fitted parameter b+1 as the individual growth rate. The fitted b for every mutant served as a ranking indicator, by which the slowest-growing and fastest-growing mutants were identified. This model was designed to compare the relative abundance changes of all the mutant strains in the same mixture along a time course, not to compare the relative abundance change of the same strain between different experimental conditions.

Giaever et al. collected and hybridized 10-15 mutant mixture samples under the control condition for each generation time [2]. For each generation time, a Gaussian distribution was fitted to the base-10 logarithm of the signal intensity for each tag across all control hybridizations. For each signal in treated condition, the likelihood of observing it under the control distribution was calculated. The fitness of a strain is then found by averaging the likelihood of the four PM (*uptag-PM, uptag-cPM, downtag-PM*, and *downtag-cPM*) signals associated with that strain. In a subsequent study, Giaever et al. made slight changes to this method by combining the four PM signals into a unified signal before fitting the Gaussian distribution [3]. Lum et al. put mutant mixture into many experimental conditions [4]. They identified mutants that are sensitive to every experimental condition, using all the rest experimental conditions as control. Similar to Giaever’s method, they judged the difference of fitness by the mean and variance of the treated signal and the “pooled control” signals. These methods worked well for the comparison between one treated sample and multiple control samples for each time point. These methods cannot be directly applied if multiple arrays are used to measure the mutants under treatment.

Tusher et al. introduced the false discovery rate (FDR) idea to determine the significance of gene expression changes while accounting for the enormous number of genes [5]. Birrell et al. showed Tusher’s methodology could be introduced into tag array analysis under a simple setting [6]. In their setting, the treatment was applied at a single dosage, and all data were obtained at a single time point. They averaged the four probe signals to get one unified signal in data preprocessing step. With three replicate samples, for every mutant they obtained three signals under each experimental condition. They applied the SAM software [5] to calculate the FDR for each mutant, and identified the most sensitive mutants to the treatment. We [7] expanded Birrell’s experimental design into treatment with multiple dosages and measuring at multiple time points.

References

1. Winzeler, E.A., et al., Functional characterization of the S. cerevisiae genome by gene deletion and parallel analysis. Science, 1999. 285(5429): p. 901-6.

2. Giaever, G., et al., Functional profiling of the Saccharomyces cerevisiae genome. Nature, 2002. 418(6896): p. 387-91.

3. Giaever, G., et al., Chemogenomic profiling: identifying the functional interactions of small molecules in yeast. Proc Natl Acad Sci U S A, 2004. 101(3): p. 793-8.

4. Lum, P.Y., et al., Discovering modes of action for therapeutic compounds using a genome-wide screen of yeast heterozygotes. Cell, 2004. 116(1): p. 121-37.

5. Tusher, V.G., R. Tibshirani, and G. Chu, Significance analysis of microarrays applied to the ionizing radiation response. Proc Natl Acad Sci U S A, 2001. 98(9): p. 5116-21.

6. Birrell, G.W., et al., A genome-wide screen in Saccharomyces cerevisiae for genes affecting UV radiation sensitivity. Proc Natl Acad Sci U S A, 2001. 98(22): p. 12608-13.

7. Dorer, R.K., et al., A small-molecule inhibitor of Mps1 blocks the spindle-checkpoint response to a lack of tension on mitotic chromosomes. Curr Biol, 2005. 15(11): p. 1070-6.
