# Supplementary material for: TagSmart: analysis and visualization for yeast mutant fitness data measured by tag microarrays
Source: BMC Bioinformatics. 2007 Apr 18;8:128. doi: 10.1186/1471-2105-8-128 (PMC1868768; doi:10.1186/1471-2105-8-128)

Figure S2: Screenshot of the preprocessor module (Windows version. Linux and Mac versions are in command line format). Both CEL files and TXT files are allowed. The user should indicate the appropriate file format in the drop down menu. The user can check the “Bad tag filtering” checkbox to eliminate bad tags from subsequent analysis.


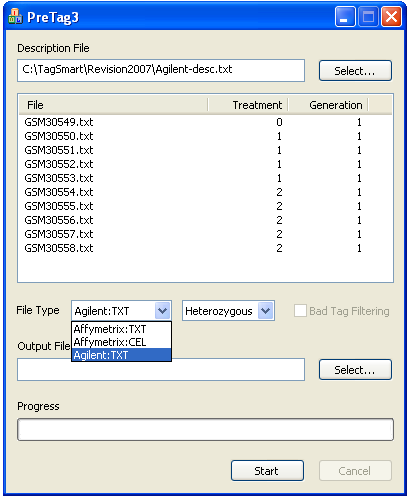

Supplement: Additional file 3 — Screenshot of the preprocessor module. Supplementary figure 2 [file 1471-2105-8-128-S3.doc]
