# Supplementary figures and images for: TagSmart: analysis and visualization for yeast mutant fitness data measured by tag microarrays
Source: BMC Bioinformatics. 2007 Apr 18;8:128. doi: 10.1186/1471-2105-8-128 (PMC1868768; doi:10.1186/1471-2105-8-128)

Figure S3: Screenshot of the computation module.

**
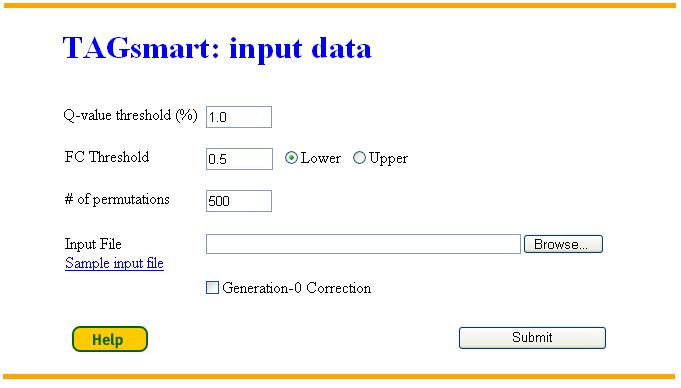
**

Supplement: Additional file 4 — Screenshot of the computation module. Supplementary figure 3 [file 1471-2105-8-128-S4.doc]
