# Supplementary material for: TagSmart: analysis and visualization for yeast mutant fitness data measured by tag microarrays
Source: BMC Bioinformatics. 2007 Apr 18;8:128. doi: 10.1186/1471-2105-8-128 (PMC1868768; doi:10.1186/1471-2105-8-128)

Figure S4: An example text output from TagSmart server. Data can be sorted by each column, with a click on the column name. More output data can be assessed by clicking the page numbers at the bottom of this webpage. More information can be downloaded as a text file by clicking the disk icon on the top of this page.


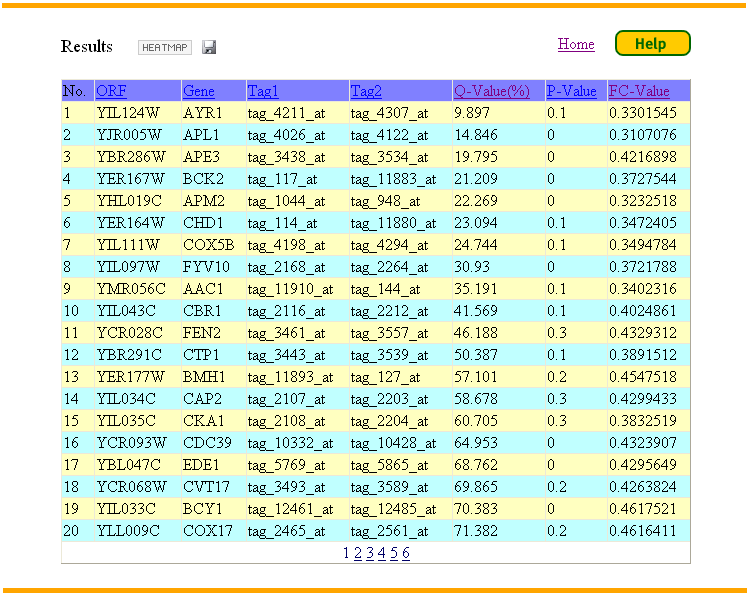

Supplement: Additional file 5 — An example text output from TagSmart server. Supplementary figure 4 [file 1471-2105-8-128-S5.doc]
