# Supplementary material for: TagSmart: analysis and visualization for yeast mutant fitness data measured by tag microarrays
Source: BMC Bioinformatics. 2007 Apr 18;8:128. doi: 10.1186/1471-2105-8-128 (PMC1868768; doi:10.1186/1471-2105-8-128)
Supplement: Additional file 6 — Summary of the Cincreasin dataset. Supplementary table 1 [file 1471-2105-8-128-S6.doc]

Table S1: Summary of the Cincreasin dataset. The columns are the experimental conditions. The rows are cell generations (time). The number of tag arrays applied under each condition is listed. At time-0, the mutant population is assumed the same for all mutants, and therefore there is no difference among experimental conditions.

| 0 generation | 5 | | | |
| --- | --- | --- | --- | --- |
|  | Control | 100 uM trt. | 200 uM trt. | 400 uM trt. |
| 4 generation | 6 | 5 | 5 | 5 |
| 8 generation | 6 | 5 | 5 | 5 |
| 16 generation | 6 | 5 | 5 | 5 |
