# Supplementary material for: TagSmart: analysis and visualization for yeast mutant fitness data measured by tag microarrays
Source: BMC Bioinformatics. 2007 Apr 18;8:128. doi: 10.1186/1471-2105-8-128 (PMC1868768; doi:10.1186/1471-2105-8-128)
Supplement: Additional file 7 — Summary of test results by colonial assays. Supplementary table 2 [file 1471-2105-8-128-S7.doc]

**Table S2**: Summary of test results by colonial assays. The “location” column gives IDs that correspond to the IDs showing up in Figure S4 (Additional file 9). The sensitivity of a mutant to Cincreasin in the colonial assay is reported in the “retest result” column. The Gene Ontology term IDs associated with each gene is given in the “gene ontology” column.

| ORF | gene | location | retest result | gene ontology |
| --- | --- | --- | --- | --- |
| WT-1 |  |  | not sensitive |  |
| WT-2 |  |  | not sensitive |  |
| WT-3 |  |  | not sensitive |  |
| YLR090W | XDJ1 | 203 B12 | sensitive | 0000723; 0005739; 0005634; 0030188 |
| YHL036W | MUP3 | 216 H2 | resistant | 0006865; 0016020; 0015191 |
| YHL019C | APM2 | 217 A6 | sensitive | 0016192; 0030121; 0030276 |
| YHL007C | STE20 | 217 B4 | sensitive | 0000282; 0001403; 0000750; 0006468; 0007124; 0007096; 0000131; 0043332; 0004674 |
| YOR288C | MPD1 | 224 H10 | sensitive | 0006457; 0000324; 0003756; 0015035 |
| YIL012W |  | 237 A8 | sensitive |  |
| YIL017C | VID28 | 237 B1 | very sensitive | 0045721; 0005737; 0005634; 0005554 |
| YIL023C |  | 237 B5 | sensitive | 0000004; 0008372; 0005554 |
| YIL045W | PIG2 | 237 C10 | sensitive | 0005979; 0005737; 0008599 |
| YIL035C | CKA1 | 237 C2 | sensitive | 0006873; 0030468; 0000501; 0000082; 0000086; 0006468; 0006356; 0006359; 0006974; 0005956; 0004682 |
| YIL096C |  | 237 E11 | sensitive | 0007046; 0005730; 0005634; 0005554 |
| YIL078W | THS1 | 237 E2 | sensitive | 0006412; 0005737; 0005739; 0004829 |
| YIL084C | SDS3 | 237 E4 | sensitive | 0006342; 0016575; 0000118 |
| YIL093C | RSM25 | 237 E9 | sensitive | 0043037; 0005763; 0003735 |
| YFL037W | TUB2 | 237 H1 | very sensitive | 0045143; 0000070; 0000743; 0030473; 0005881; 0005828; 0005880; 0005827; 0005816; 0045298; 0005200 |
| YGR264C | MES1 | 238 D5 | sensitive | 0006431; 0005737; 0017102; 0004825 |
| YDL229W | SSB1 | 246 F8 | sensitive | 0051083; 0006412; 0006450; 0005844; 0005625; 0016887; 0051082 |
| YDL234C | GYP7 | 246 G1 | sensitive | 0016192; 0005737; 0005097 |
| YDR018C |  | 247 A3 | sensitive | 0008654; 0008372; 0008415 |
| YJL015C |  | 248 A10 | sensitive |  |
| YJL020C |  | 248 B3 | sensitive | 0030036; 0030479; 0017024 |
| YJR005W | APL1 | 248 C4 | sensitive | 0016192; 0030122; 0005554 |
| YJR045C | SSC1 | 248 F3 | sensitive | 0006457; 0030150; 0042026; 0005743; 0042645; 0005739; 0001405; 0016887; 0030234; 0008565 |
| YIL008W | URM1 | 250 B9 | sensitive | 0007114; 0001403; 0006464; 0006979; 0005737; 0005634; 0031386 |
| YIL059C |  | 250 C10 | sensitive |  |
| YIL047C | SYG1 | 250 C6 | sensitive | 0007165; 0005739; 0005886; 0005554 |
| YIL055C |  | 250 C9 | sensitive | 0000004; 0008372; 0005554 |
| YIL069C | RPS24B | 250 D4 | sensitive | 0043037; 0005843; 0003735 |
| YIL112W | HOS4 | 250 F11 | sensitive | 0016575; 0045835; 0000118; 0017136; 0045129 |
| YIL105C |  | 250 F5 | very sensitive | 0045011; 0030036; 0030950; 0001558; 0005737; 0005739; 0005886; 0031932; 0035091 |
| YIL107C | PFK26 | 250 F7 | very sensitive | 0006003; 0006110; 0005737; 0003873 |
| YIL124W | AYR1 | 250 G9 | sensitive | 0006654; 0005737; 0005783; 0005811; 0005741; 0005739; 0000140 |
| YIL141W |  | 250 H11 | sensitive |  |
| YIL135C |  | 250 H6 | sensitive | 0000082; 0005737; 0005554 |
| YIL137C |  | 250 H7 | sensitive | 0006508; 0005737; 0004222 |
| YIL153W | RRD1 | 251 A10 | sensitive | 0006281; 0030472; 0006970; 0000267; 0005737; 0005634; 0008601 |
| YIL167W |  | 251 B11 | sensitive | 0009069; 0008372; 0003941 |
| YIL160C | POT1 | 251 B4 | sensitive | 0006635; 0005782; 0003988 |
| YIL161W |  | 251 B5 | very sensitive | 0000004; 0005737; 0005554 |
| YIL165C |  | 251 B9 | sensitive | 0000004; 0008372; 0005554 |
| YIR008C | PRI1 | 251 C10 | sensitive | 0006260; 0006270; 0006269; 0006273; 0005658; 0003896; |
| YIL171W | HXT12 | 251 C2 | sensitive | 0000004; 0008372; 0005554 |
| YDL028C | MPS1 | 258 F1 | sensitive | 0051227; 0007094; 0007103; 0000778; 0005816; 0004712 |
| YGL121C | GPG1 | 260 D12 | sensitive | 0007165; 0008372; 0004871 |
| YGL112C | TAF60 | 260 D4 | sensitive | 0016568; 0006325; 0000114; 0016573; 0006473; 0006367; 0000124; 0046695; 0005669; 0016251 |
| YGL146C |  | 260 F11 | sensitive | 0000004; 0016020; 0005554 |
| YER164W | CHD1 | 261 F9 | sensitive | 0006338; 0006368; 0005739; 0000124; 0046695; 0008023; 0016887; 0016944 |
| YER178W | PDA1 | 261 G11 | sensitive | 0006090; 0042645; 0005739; 0005967; 0004739 |
| YMR054W | STV1 | 262 A2 | sensitive | 0007035; 0005794; 0000220; 0005770; 0046961 |
| YMR056C | AAC1 | 262 A4 | sensitive | 0009060; 0006839; 0005743; 0005471 |
| YIL158W |  | 262 B6 | sensitive | 0000324; 0000004; 0005554 |
| YIL111W | COX5B | 262A12 | sensitive | 0006123; 0005751; 0004129 |
| YIL051C | MMD1 | 262B9 | very sensitive | 0009097; 0000002; 0005759; 0005739; 0005554 |
