# Supplementary material for: TagSmart: analysis and visualization for yeast mutant fitness data measured by tag microarrays
Source: BMC Bioinformatics. 2007 Apr 18;8:128. doi: 10.1186/1471-2105-8-128 (PMC1868768; doi:10.1186/1471-2105-8-128)
Supplement: Additional file 8 — Fold changes of the top 10 mutants sensitive to Cincreasin treatment. Supplementary figure 5 [file 1471-2105-8-128-S8.doc]

**Figure S5**: Fold changes of the top 10 mutants sensitive to Cincreasin treatment.
