# Supplementary material for: TagSmart: analysis and visualization for yeast mutant fitness data measured by tag microarrays
Source: BMC Bioinformatics. 2007 Apr 18;8:128. doi: 10.1186/1471-2105-8-128 (PMC1868768; doi:10.1186/1471-2105-8-128)
Supplement: Additional file 9 — Colonial assays for testing S.cerevisiae mutants in the presence of DMSO (control) and Cincreasin (treatment). Supplementary figure 6 [file 1471-2105-8-128-S9.doc]

**Figure S6**: Colonial assays for testing S.cerevisiae mutants in the presence of DMSO (control) and Cincreasin (200uM and 400uM). Some mutant names are coded by a “location” number. This number corresponds to the number in the “location” column in Table S2. The mutant names are available in Table S2 (Additional file 7). Every mutant occupies four consecutive colonies in a row. Two different mutants occupy a complete row in a plate. For example, mutant 217A6 occupies the four left colonies in the first row in the first plate. Mutant 237C10 occupies the four right colonies in the same row. The three plates in the same row has the same arrangement of mutant colonies, therefore the mutant “location” number is only given to the left plate. For each mutant, the four colonies from left to right are at their 0, 4, 8, and 16 cell generations, respectively. The difference of fitness for a mutant and be obtained by comparing the sizes of the colonies across the colonies at the same location of the three plates on the same row. The test result is given in Table S2.

**
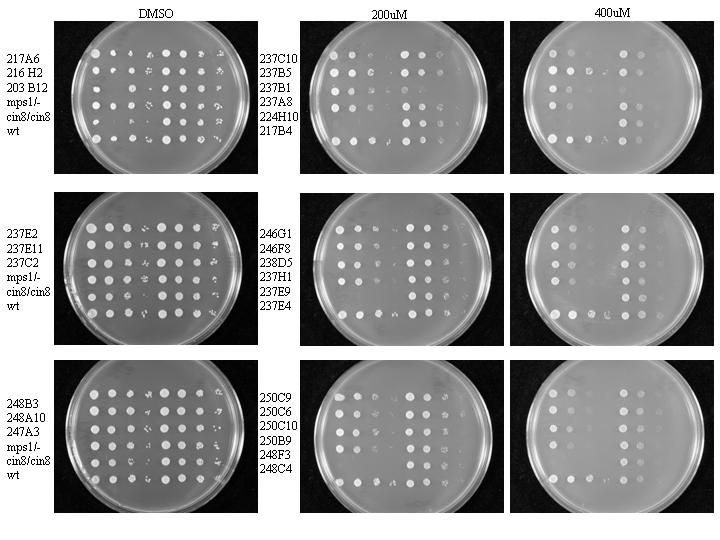
**

**
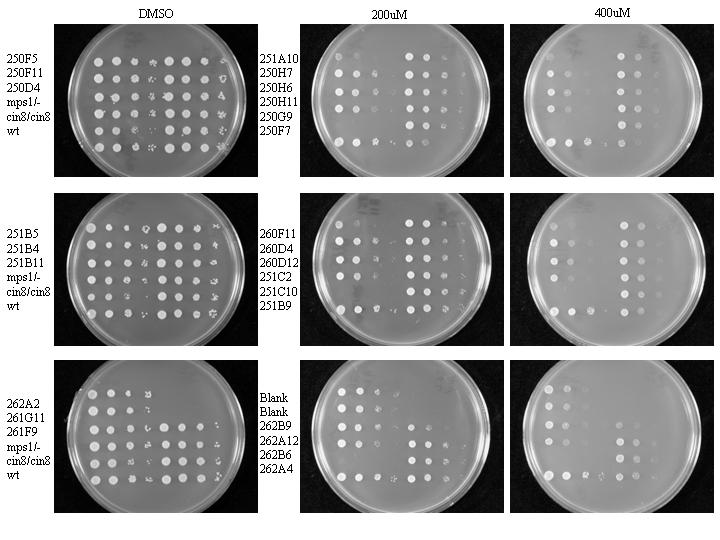
**
